# Supplementary material for: Patterns of Intron Gain and Loss in Fungi
Source: PLoS Biol. 2004 Nov 30;2(12):e422. doi: 10.1371/journal.pbio.0020422 (PMC532390; doi:10.1371/journal.pbio.0020422)
Supplement: Table S1 — Also available at http://genes.mit.edu/NielsenEtAl/. (4.3 MB ZIP). [file pbio.0020422.st001.zip › NielsenEtAl/html/26.html]

AN5832.1.NCU03616.1.MG09499.1.FG10114.1


```
 CLUSTAL W (1.82) Multiple Sequence Alignments - Introns Inserted


Sequence 1: MG09499.1	241 aa
Sequence 2: FG10114.1	235 aa
Sequence 3: NCU03616.1	229 aa
Sequence 4: AN5832.1	225 aa
Alignment Length: 250 aa
Number Identitical Residues: 136 aa
Alignment Score (without introns) 5802


MG09499.1 	MTG-RLQLHKLVVLGDGGVGKTALTIQ~LCLQHFVET0YDPTIEDSYRKQVVIDNQACML
NCU03616.1	MVGNKQVLYKLVVLGDGGVGKTALTIQ~LCLEHFVET0YDPTIEDSYRKQVVIDGQACML
FG10114.1 	MAG-RMVLYKLVVLGDGGVGKTALTIQ~LCLQHFVET0YDPTIEDSYRKQVVIDGQPCML
AN5832.1  	MAG-KMTLYKLVVLGDGGVGKTALTIQ0LCLNHFVET~YDPTIEDSYRKQVVIDQQSCML
          	*.* :  *:****************** ***:***** **************** *.***

MG09499.1 	EVLDTAGQEEYTALRDQWIRDGEGFVLVYSISSRSSFSRIKRFHHQIQRVKESCASSP--
NCU03616.1	EVLDTAGQEEYTALRDQWIRDGEGFVLVYSISSRSSFARIKKFHHQIQRVKESTSSPS--
FG10114.1 	EVLDTAGQEEYTALRDQWIRDGEGFVLVYSISSRSSFTRIKRFHHQIQRVKESCASSP--
AN5832.1  	EVLDTAGQEEYTALRDQWIRDGEGFVLVYSITSRASFSRITKFYNQIKMVKESANSSSPS
          	*******************************:**:**:**.:*::**: ****  *...:

MG09499.1 	--SYPGS-PIATVT-TQAPVPIMLVGNKSDRVTEREVSTQEGHALARELGCEFVEASAKN
NCU03616.1	--AYPGSSPLAATN-PSAPVPIMLVGNKSDRVTEREVSTQEGHALARELGCEFTEASAKT
FG10114.1 	--SYPGS-PISSAN-PQLPVPIMLVGNKSDRVTEREVSTQEGHALARELGCEFVEASAKN
AN5832.1  	GASYLGS-PMSSPSGPPLPVPVMLVGNKSDKAVERAVSAQEGQALAKDLGCEFVEASAKN
          	.::* ** *::: ...  ***:********:..** **:***:***::*****.*****.

MG09499.1 	CIN~VEKAFYDVVRILRRQRQQ-ASRP-SLPGNSRTKTGGMGKSESFYQSDGKRGSRKDG
NCU03616.1	RTN~VEKAFYDVVKQLRKQRQQGQSTPRALPPSGNSKSE---KYSGTEKPKRPRG-----
FG10114.1 	CIN~VEKAFYDVVRILRRQRQQ-ASRP-SDRSSGRTRTG-NGDARGGDRDERHRNRNKDR
AN5832.1  	CIN0----------RMQQQRPQDTRRTTGLGQMRDSGPE----YPRTFRPDRPRHRGG--
          	  *            :::** *.   . .      : .          : .  *      

MG09499.1 	EKHRSKPIKCVIL
NCU03616.1	--------KCLII
FG10114.1 	NKS-----KCVVL
AN5832.1  	-------IKCQIL
          	        ** ::
```
